# Elasticity of polymeric nanocolloidal particles

**DOI:** 10.1038/srep15854

**Published:** 2015-11-02

**Authors:** Jonas Riest, Labrini Athanasopoulou, Sergei A. Egorov, Christos N. Likos, Primož Ziherl

**Affiliations:** 1 Faculty of Physics, University of Vienna, Boltzmanngasse 5, A-1090 Vienna, Austria; 2 Forschungszentrum Jülich GmbH, ICS-3—Soft Condensed Matter, Leo-Brandt-Straße, D-52425 Jülich, Germany; 3 Jožef Stefan Institute, Jamova 39, SI-1000 Ljubljana, Slovenia; 4 Department of Chemistry, University of Virginia, McCormick Road, Charlottesville, VA 22904-4319, USA; 5 Faculty of Mathematics and Physics, University of Ljubljana, Jadranska 19, SI-1000 Ljubljana, Slovenia; 6 Erwin Schrödinger International Institute for Mathematical Physics, University of Vienna, Boltzmanngasse 9, A-1090 Vienna, Austria

## Abstract

Softness is an essential mechanical feature of macromolecular particles such as polymer-grafted nanocolloids, polyelectrolyte networks, cross-linked microgels as well as block copolymer and dendrimer micelles. Elasticity of individual particles directly controls their swelling, wetting, and adsorption behaviour, their aggregation and self-assembly as well as structural and rheological properties of suspensions. Here we use numerical simulations and self-consistent field theory to study the deformation behaviour of a single spherical polymer brush upon diametral compression. We observe a universal response, which is rationalised using scaling arguments and interpreted in terms of two coarse-grained models. At small and intermediate compressions the deformation can be accurately reproduced by modelling the brush as a liquid drop, whereas at large compressions the brush behaves as a soft ball. Applicable far beyond the pairwise-additive small-strain regime, the models may be used to describe microelasticity of nanocolloids in severe confinement including dense disordered and crystalline phases.

Mechanical properties are often among the most attractive aspects of both synthetic and natural nanoparticles. In some cases, the nanoparticles stand out because of extraordinary stiffness and strength, and in others elasticity controls their shape, interactions, or self-assembly. Much of the observed behaviour is consistent with macroscopic continuum elasticity despite the small particle size. For example, the rippled shape of bent carbon nanotubes^1^ as well as the deformation of microtubules^2^ and protein nanotubes^3^ indented by a sharp tip can be explained in terms of thin shell theory and so can be the faceted icosahedral form of large spherical viral capsids^4^. Also within the domain of classical elasticity are the Hertz theory used to interpret the correlations in solutions of microgels^5^ and the indentation of polymer microspheres with nanosize tips^6^ as well as models of adhesion and wetting of hard^7^,^8^ and soft nanoparticles^9^,^10^,^11^.

The applicability of continuum elasticity at such small scales is far from obvious and must be validated by simulations^12^,^13^,^14^ which, among other things, point to the importance of surface elastic terms needed to account for the size dependence of the moduli^12^. The agreement found is generally rather good, encouraging further use of the continuum description. A particularly interesting area is the mechanics of nanocolloids such as polymer-grafted nanoparticles and dendrimer micelles, where elastic theory can be employed to construct coarse-grained models^15^ capable of capturing the many-body effects^16^.

We explore this idea by probing the elasticity of spherical polymer brushes (SPBs; Fig. 1a) which interpolate between star polymers^17^ and polymer-stabilised colloids. Using simulations and self-consistent field theory, we study static deformations of a single SPB in good solvent conditions confined to a slit and perform diametral compression as a standard mechanical test^18^. The results are explained in terms of two complementary models, suggesting that the SPB behaves as a liquid drop at small compressions and solidifies at large compressions. Furthermore, we use scaling arguments to explain the universal deformation response virtually independent on the number and length of chains in the SPB. Our predictions can be explored experimentally either directly in force-deformation measurements of SPBs^19^ or indirectly by analysing the structure of their condensed phases and aggregates^20^.

## Results

**Figure 1:**
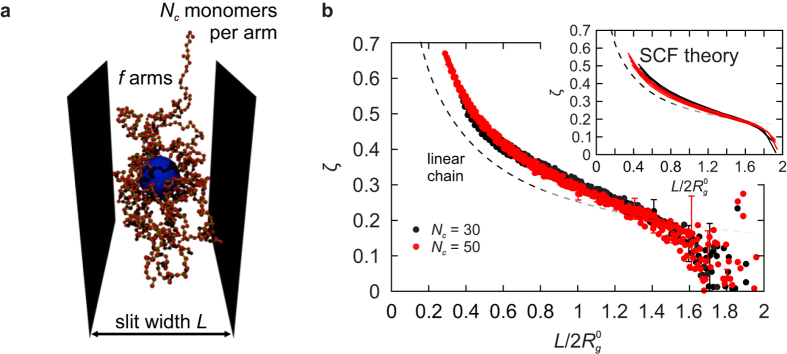
Universal deformation behaviour of SPBs. (**a**) Simulation snapshot of an SPB illustrating the geometry studied; monomers are not plotted to scale for clarity. (**b**) MD reduced central lateral extension 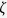 vs. reduced slit width 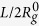 for thin (*N*_*c*_ = 30, black circles) and thick (*N*_*c*_ = 50, red circles) SPBs of functionalities *f* = 30, 40, 50, and 60. For clarity, only a few representative errorbars are shown. The scaling-theory 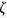 of a linear chain [equation (2)] is plotted with the dashed line faded at 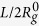  1.5 to emphasize that it is valid only in narrow slits. Inset: 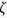 obtained from SCF theory for the same *f* and *N*_*c*_ plotted using the same color code; also included is equation (2) (dashed line).

We use molecular dynamics (MD) simulations to study the deformation of an SPB consisting of a small hard colloidal particle grafted with a polymer brush of *f* linear-chain arms each consisting of *N*_*c*_ monomers; temperature was fixed at 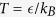 where 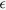 is the energy scale of the monomer-monomer repulsion (see Methods). The SPB is confined to a slit formed by parallel immobile walls (Fig. 1a) and its deformation is quantified by the ratio of central lateral extension and indentation denoted by *ζ*. To evaluate it, we computed the dimensions of the SPB based on monomer densities projected on the axes of the coordinate system centered at the SPB and oriented such that the *z* axis is perpendicular to the walls; note that the projected densities 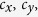 and 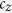 are measured in units of 1/length rather than in units of 1/volume as the usual monomer density *c*. SPB half-thickness is given by the transverse semiaxis 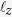 defined by 
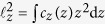
 and the in-plane semiaxes 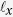 and 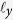 corresponding to the waist radius are introduced analogously. The reduced central lateral extension then reads


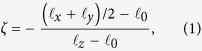


where 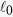 is the radius of an isolated SPB defined by 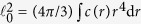 evaluated in absence of walls. We find that our 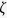 is more meaningful than its analogue based on the eigenvalues of the radius of gyration tensor, which carries a larger numerical error at small compressions.

Figure 1b shows the reduced central lateral extension 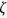 for thin (*N*_*c*_ = 30) and thick (*N*_*c*_ = 50) SPBs with functionalities *f* = 30, 40, 50, and 60; the screening length 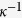 of the Yukawa wall potential is a small fraction of the radius of gyration of an isolated SPB 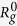 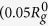 in the thin and 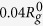 in the thick SPB). The eight datasets plotted against reduced slit width 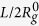 collapse surprisingly well despite considerable variation of chain length *N*_*c*_ and functionality *f*, and they reveal three distinct deformation regimes.

**Figure 2:**
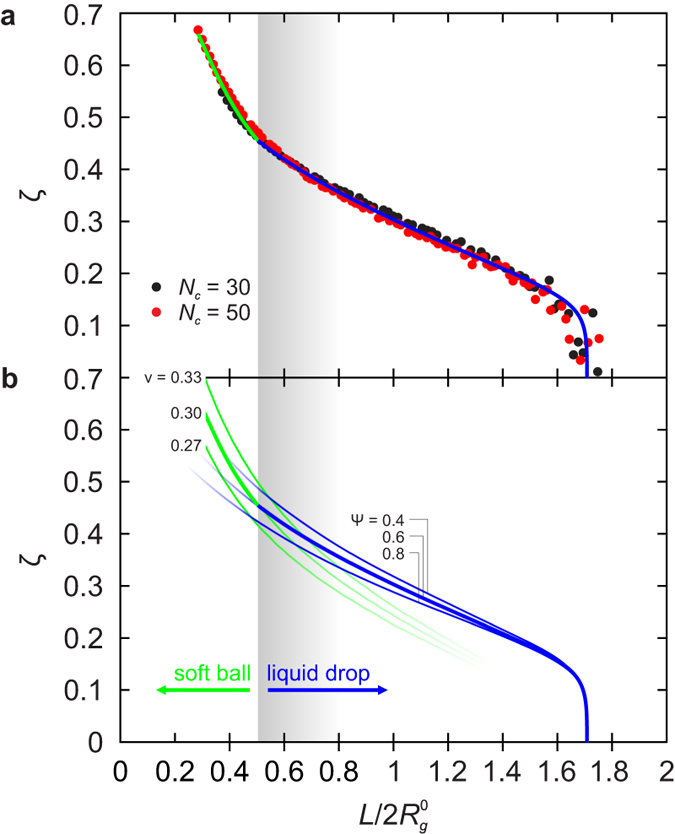
Liquid-drop/soft-ball fit. (**a**) Reduced central lateral extension of *N*_*c*_ = 30 and *N*_*c*_ = 50 *f* = 60 SPB (circles) and the best fit combining the 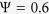 liquid-drop model at 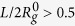 (blue line) with 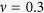 soft-ball model at 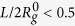 (green line). (**b**) Liquid-drop 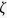 for 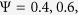 and 0.8 (blue lines) and the soft-ball 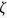 for 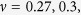 and 0.3 (green lines). The former does not reproduce the MD results at small 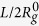  0.5 even if 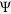 departs from the best-fit value of 0.6, and the latter predicts too small a central lateral extension in the small- and the intermediate-deformation regime irrespective of the value of 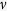. Each set of curves is faded at reduced slit widths where the respective model does not apply. The best-fit curves from panel a are replotted with thick lines.

At small compressions the MD results are rather scattered due to smallness of both numerator and denominator in equation (1) but they still reveal a knee-like increase of 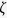 clearly visible in the two *f* = 60 datasets replotted in Fig. 2a. This behaviour is additionally confirmed by the self-consistent field theory (SCF; see Methods). SCF results shown in the inset to Fig. 1b agree well with the MD data but are considerably less noisy, emphasising the knee-like onset of deformation.

The narrow small-compression regime is followed by a broad, virtually linear variation of 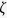 extending from reduced slit width 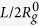 of about 1.6 down to about 0.7. At 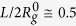, the SPBs undergo a transition to the large-compression regime characterised by a steep increase of 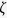 upon compression.

The effective SPB diameter can be defined by the onset of deformation where the slit serves as a vernier caliper. The *f* = 60 MD data in Fig. 2a show that 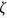 is nonzero at 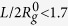 so that the effective diameter is 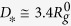 whereas the SCF theory puts it at about 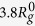. These values are consistent with experiments on linear polymers such as DNA. A single DNA molecule is affected by confinement^21^ at 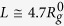; this is the effective diameter. Since a linear polymer is more anisometric than an SPB, it must have a larger ratio 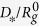 so that our estimates are reasonable. Finally, the auxiliary Yukawa potential used in simulations introduces an effective slit width smaller than the nominal *L* by about 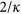. Thus the true diameters of the (*N*_*c*_ = 30) and the (*N*_*c*_ = 50) SPB are smaller than the above D_*_ by about 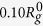 and 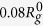, respectively.

The remarkable universality of the SPB deformation must stem from a basic feature of polymers, and it is instructive to begin understanding it by a scaling-theory estimate of 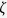 for a single linear chain. In severe confinement^22^, the in-plane diameter of the chain is 
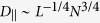
 whereas its transverse size is 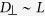, which gives


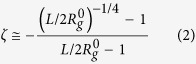


irrespective of chain length. The dashed line in Fig. 1b shows that this result is in good semiquantitative agreement with the MD data although it relies on the blob picture of a linear chain with excluded-volume interactions rather than on the geometrically more involved blob analysis of the deformation of a multiarm brush^23^. The agreement suggests that it is worthwhile seeking a coarse-grained interpretation that does not depend on the details of the macromolecular architecture, and here we propose two complementary continuum theories.

### Liquid-drop model

The small- and the intermediate-compression regimes are captured very well by a model where the SPB is viewed as a liquid drop of volume *V* and area *A* characterised by a phenomenological free energy


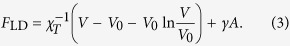


Here *γ* is the surface tension and *V*_0_ is the reference volume where the pressure within the drop given by the Murnaghan equation of state^24^ 
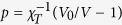
 vanishes in absence of surface tension so that 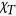 is the isothermal compressibility of the drop at *p* = 0 and *γ* = 0. First proposed for hydrostatic compression of elemental substances^24^ and previously considered in models of compressible rubber^25^, this equation captures the essential physics of simple fluids. The ideal-gas-like term ensures that the pressure diverges at small volumes, thereby phenomenologically accounting for the repulsive interparticle forces, whereas the negative, volume-independent term represents the effect of the cohesive interactions.

Equation (3) contains a single surface term although it could be split into two contributions corresponding to the free surface of the brush and to the brush-wall contact zone. Such a generalization is justified even in inert walls studied here, yet we stick to the more transparent single-surface-tension variant of the model because it already offers a very accurate interpretation of the shape of the confined SPB as shown below. Within this model, the deformation of the drop is controlled by a single dimensionless parameter


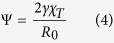


equal to twice the ratio of the Egelstaff-Widom length^26^ 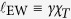 and the reference drop radius 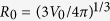.

The reference volume *V*_0_ can be thought of as a spherical region of the liquid of radius *R*_0_ far from any confining walls, which is surrounded by a mathematical surface without tension. Once this sphere is endowed with surface tension *γ*, its radius shrinks from *R*_0_ to a new value 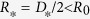, giving rise to a Laplace pressure difference across the surface. From equation (3), it is straightforward to evaluate the shrinking factor 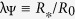 by minimizing *F*_LD_ for spherical shapes, yielding


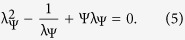


For an incompressible fluid where 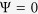, the drop does not shrink at all and 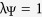. It follows that 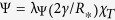, the term in the parenthesis representing the Laplace pressure. Unlike in ordinary liquids, in which the drop size R_*_ can be arbitrarily large, for spherical polymer brushes R_*_ is a length-scale determined by the brush architecture, *f* and *N*_*c*_. Accordingly, it is physically meaningful to treat Ψ rather than 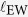 as an intrinsic material parameter, and we refer to it as the *reduced Egelstaff-Widom length*. It is also convenient to define the deformation free energy, 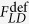, as the difference between the liquid-drop free energy of a confined drop and that of a free drop of radius R_*_. From equation (3) we obtain


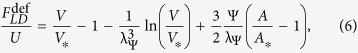


where V_*_ and A_*_ are the volume and surface area of the drop of radius R_*_, respectively, and the energy scale *U* is given by


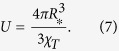


Note that in this way, the reference lengthscale *R*_0_ has completely dropped out as it should. The model is fully described by two parameters, the reduced Egelstaff-Widom length Ψ which sets the shape of the deformation and *U* which sets the overall scale of the energy penalty to compress the drop.

In Fig. 2a we compare the *f* = 60 MD data to the Ψ = 0.6 liquid-drop 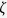 calculated from the semiaxes of the drop which were obtained numerically using Surface Evolver^27^ (see Methods). The model reproduces very well the small- and the intermediate-compression regimes at slit widths ranging from 100% down to about 30% of the effective diameter 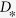. Note that 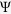 is essentially the sole fitting parameter as the MD data are consistent only with very limited variations of 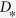; here we used 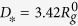.

In the large-compression regime the model is no longer suitable. This is shown by the continuation of the best-fit 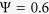 liquid-drop 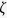 at small slit widths 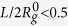 plotted in Fig. 2b, which underestimates waist extension increasingly more as *L* is decreased and leads us to the condition that in this regime the behaviour of the SPB is qualitatively different. Figure 2b also demonstrates that the small spread of the eight MD datasets in Fig. 1b in the small- and intermediate-deformation regime can be associated with slight variations of Ψ—an order of magnitude smaller than those shown in Fig. 2b.

### Soft-ball model

To reproduce the deformation at large compressions, we turn to an alternative model where the SPB is represented by an elastic solid sphere. The underlying rationale is that in the large-compression regime, chain fluctuations are reduced considerably by a combination of topological restrictions due to grafting and confinement, implying that the brush may well behave effectively as a solid rather than as a liquid.

Among the several types of elasticity of isotropic media, we choose the modified neo-Hookean theory originating in the statistical thermodynamics of a three-dimensional polymer network^28^. The corresponding free energy density reads^29^


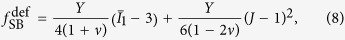


where *Y* is the Young modulus and 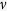 is the Poisson ratio whereas 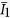 is the first invariant of the isochoric part of the Green deformation tensor 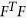, *F* being the deformation gradient, and 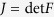. Like in the liquid-drop model, it would be reasonable to complement the soft-ball elastic energy by the surface energy. Yet within the minimal framework this is not needed because shear elasticity alone resists shape deformation. To keep the model as simple as possible, we opt to not include the surface tension so that the ball shape is controlled solely by the Poisson ratio 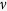, whereas the deformation energy scales as 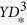.

The equilibrium shape of the ball at a given *L* was found by minimizing the elastic energy using the FreeFEM++ package^30^ (see Methods). We varied the Poisson ratio so as to obtain an optimal agreement at large compressions, finding that the best-fit value of 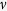 is 0.3 (Fig. 2a); deviations not exceeding 0.01 are sufficient to explain the small variations of 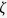 across the datasets (Fig. 2b). The failure of the soft-ball model in the small- and intermediate-deformation regime, especially at the onset of deformation at 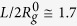, can be understood by visualising a slightly compressed sponge ball where stresses due to compression are localized right at the two walls and do not reach into the bulk, and the waist diameter is thus barely increased. In a liquid drop, on the other hand, any increase of pressure upon compression is communicated across all of the volume and thus the waist extension relative to compression is considerable even at small compressions.

### Density profiles

**Figure 3:**
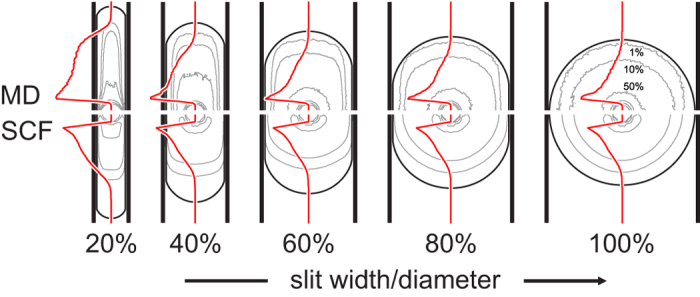
Shape of deformed SPB. Gray lines show the monomer density isolines corresponding to 50%, 10%, and 1% of the maximal density of the thick *N*_*c*_ = 50, *f* = 60 SPB at five relative slit widths, and red curves are midplane radial profiles. Top and bottom halves shows MD and SCF results, respectively. Also plotted are the theoretical SPB contours of the combined soft-ball/liquid-drop model (solid black lines); the transition is at 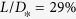.

Our theoretical description of the MD shape deformation data combines the predictions of the two models, the liquid-drop/soft-ball transition being at 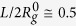. A more detailed insight into the transition as well as the SPB structure itself is provided by the monomer density profiles in Fig. 3 which shows the 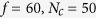 SPB in slits of width of 20%, 40%, 60%, 80%, and 100% of the effective diameter. At each slit width, we plot the density isolines at 50%, 10%, and 1% of the maximal density at that slit width as well as the density profile in the midplane; the MD and the SCF results are shown in the top and the bottom half of the panel, respectively. Compared to the MD and SCF density isolines are the surfaces of the soft-ball and the liquid-drop model below (the 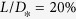 case) and above the transition (the 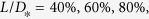 and 100% cases), respectively.

The rightmost panel in Fig. 3 shows the SPB at the onset of deformation at 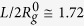 and 1.9 for the MD and the SCF results, respectively. At this slit width, the agreement of the MD and the SCF density profiles is rather good as expected^31^, the only difference being the slightly larger distance between the 1% SCF density isoline and the theoretical SPB surface which can be attributed to the fact that the effective diameter predicted by SCF is larger than that obtained by MD. The location of the transition from the inner, dense region of the SPB and the outer dilute shell with an approximately exponential density profile coincides with the theoretical surface; on the linear scale of Fig. 3, the small-magnitude exponential tail is seen as an almost straight vertical segment of the red curves. Also visible is the effect of the auxiliary Yukawa potential used in MD simulations which produces a depleted subsurface layer close to either wall, and a few artifacts of the cubic lattice used in SCF theory (say in the kidney-shaped 50% isoline).

As the SPB is compressed, the agreement of the MD and the SCF results is gradually poorer. In part, this is due to the approximate treatment of the excluded-volume interactions in the SCF theory^31^ and in part it can be related to the slightly different effective SPB diameters and to the different wall types used. This is best seen in the 20% slit-width case where the SCF midplane radial density profile is representative of the density in the whole SPB whereas the MD profile varies considerably with the distance from the midplane. Still the MD midplane profiles show that the boundary of the inner dense region of the SPB correlates with the theoretical soft-ball/liquid-drop surface. Also notable is the development of the shoulder-like density profile in very compressed SPBs signaling a qualitatively different behaviour compared to small and intermediate compressions, which is consistent with the soft-ball/liquid-drop transition.

### Deformation energy

**Figure 4:**
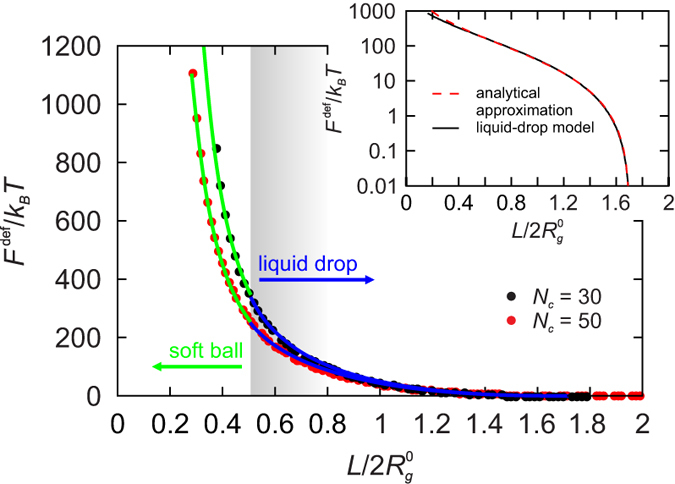
Deformation energy. Numerically computed deformation energy of the *N*_*c*_ = 30 and the *N*_*c*_ = 50 f = 60 SPB (black and red circles, respectively) and the liquid-drop and soft-ball fits (blue and green line, respectively). Inset: Liquid-drop deformation energy of the *N*_*c*_ = 50, *f* = 60 SPB and its analytical approximation 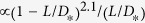, where 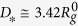.

The coarse-grained picture is completed by comparing the MD deformation energy to those of the two models. To this end, the effect of the Yukawa wall potential is taken into account by recognizing that at small slit widths 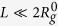 the Yukawa potential penetrates across the whole slit. The corresponding energy increase can be estimated by the magnitude of the potential in the center which is proportional to 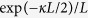. Figure 4 shows the MD energy for the *N*_*c*_ = 30 and the *N*_*c*_ = 50 *f* *=* 60 SPBs as well as the fits obtained by combining the liquid-drop and the soft-ball energies with the above Yukawa term. The agreement is very good over almost three orders of magnitude, the only systematic but insignificant deviation being the behaviour at 
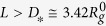
 where the liquid-drop and the soft-ball energies vanish whereas the MD energy remains finite. A close inspection reveals a minute discontinuity in the deformation energy at the liquid-drop/soft-ball transition, which may be due to the approximate treatment of the Yukawa potential.

The best fits of deformation free energy in Fig. 4 fix the characteristic energy scales of the two models, *U* [equation (7)] and 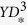. Together with the already known values of the kinematic parameters 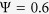 and 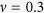 and an estimated SPB size, the energy scales can then be used to determine the liquid-drop compressibility and surface tension as well as the soft-ball Young modulus. Assuming that 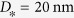, we obtain 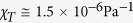, 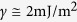, and 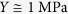 consistent with microindentation experiments on polymer nanodroplets^9^. The liquid-drop 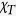 is just a little larger than the soft-ball compressibility 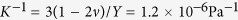 in agreement with the expectation that upon the soft-ball/liquid-drop transition, the compressibility of the SPB should not change very dramatically.

### Scaling theory

Our numerical analysis revealed a remarkable collapse of SPB deformation data. The eight datasets shown in Fig. 1b cover a broad range of experimentally relevant SPB functionalities and two chain lengths large enough so as to ensure that the reported behaviour is not affected by the monomer size. Here we provide a scaling-theory interpretation of the data collapse, which suggests that our observations are universal.

Our starting point is the des Cloizeaux formula for the osmotic pressure 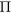 of semidilute polymer solutions, 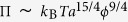, where *ϕ* is the monomer concentration and *a* is the monomer size^32^. This result can be used to calculate the reduced Egelstaff-Widom length [equation (4)] recast as 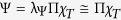 so as to emphasise that the SPB surface tension is related to the osmotic pressure by the Young-Laplace equation; here we note that for 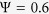, equation (5) yields 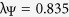, a factor of order unity which plays no role in the scaling theory. Since the colloid in the center of our SPB is small at all *N*_*c*_ and *f* studied, we can approximate the brush by a star polymer so that 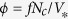, V_*_ being the star volume corresponding precisely to the volume of unconfined spherical liquid drop of radius R_*_. Thus, the osmotic pressure scales as


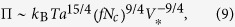


and


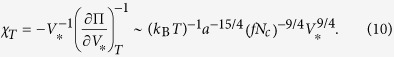


This implies that 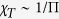 and thus the reduced Egelstaff-Widom length is a quantity of order unity that does not depend on functionality nor on chain length,


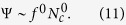


This finding is in very good agreement with the data in Fig. 1b and it establishes a universality for the shapes of starlike spherical polymer brushes under compression, independently of their functionality and chain length. Note that this prediction is valid for any power-law equation of state rather than just for the des Cloizeaux formula, and it can be attributed to the self-similar chain structure. The small deviations from the perfect data collapse may be due to the presence of the colloid because its size is the same in all SPBs studied here whereas the radius of SPB diameter varies with *N*_*c*_ and *f*.

Within the same theory, we can further extract the dependence of the energy scale *U* [equation (7)] on the brush parameters. Since 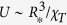, using the scaling of brush radius as^17^ 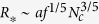 and equation (10) we readily obtain


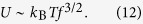


This is a very rewarding result, as it coincides with the scaling of both the pairwise effective interaction potential between star polymers^33^ and between a star and a single wall^34^. Indeed, in the limit of weak compressions, the overall deformation energy penalty paid by the brush should be pairwise additive; accordingly, the elastic theory put forward should reproduce the dependence of the interaction on *f*, as it does. Moreover, and once more in agreement with the aforementioned effective potential, there is no dependence of the energy scale on *N*_*c*_. This finding is also confirmed in our simulations: as can be seen in Fig. 2b, the deformation energy curves for the two brushes of the same *f* but different *N*_*c*_ run very close to one another for a broad range of deformations in which the liquid drop model is valid. The small deviations seen are of order 10% and can be attributed to the rigid core which makes the thinner brush effectively less compressible.

Finally, we can now provide an independent estimate of the surface tension and compressibility, based on purely theoretical arguments, and compare with the values obtained by the fit. From equations (4,7,11,12), we readily obtain the scaling laws


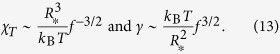


Using typical values R_*_ = 10  nm and *f* = 50, we find, at room temperature, 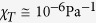 and 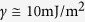, in excellent agreement with results from fits of the deformation energy.

## Discussion

The universal value of the reduced Egelstaff-Widom length 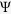 in SPBs is the most important result of the scaling theory obtained by approximating the brush by a star polymer and disregarding the hard colloidal core. Had the core been taken into account, the overall SPB compressibility and thus 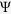 should be smaller, leading to a departure from universality. A sizable core is also expected to shift the liquid-drop/soft ball transition to narrower slits by excluding the chains from the centre where a large enough local monomer density could otherwise be reached for geometrical reasons. On the other hand, 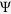 can also be controlled by intermonomer interaction and by the solvent, affecting both effective surface tension and compressibility of the SPB. Thus it seems reasonable to theoretically explore the liquid-drop model more closely for a broad range of 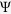 covering not only the regime directly applicable to our SPBs but also the incompressible limit at 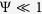 where the brush is deformed at constant volume and the tension-dominated limit at 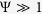 where the shape of the SPB is dictated primarily by surface tension.

**Figure 5:**
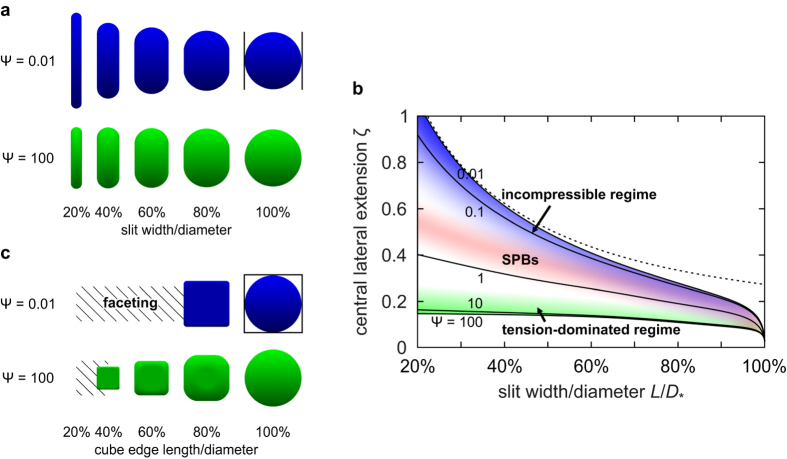
Incompressible and tension-dominated regime of the liquid-drop model. (**a**) Theoretical shapes of diametrally compressed drops with reduced Egelstaff-Widom lengths 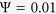 and 100 at various reduced slit widths 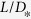: in narrow slits, the almost incompressible 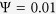 drop is deformed much more than the tension-dominated 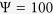 drop. (**b**) Reduced central lateral extension 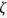 for diametrally compressed SPB of 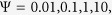 and 100. Blue and green shading represent the incompressible and the tension-dominated regimes, respectively; also shown is the SPB domain (red shading). Dashed line is the fixed-volume approximation. (**c**) Drops of 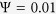 and 100 confined to a cubic cavity where the difference between the incompressible and the tension-dominated regimes are readily seen even at small compressions. As the size of the cavity is decreased, the almost incompressible 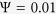 drop develops a faceted shape as soon as the cube edge length reaches about 80% of D_*_ whereas the tension-dominated drop maintains a more rounded shape down to cube edge lengths of about 40% of D_*_. In panels a and c, confining walls are only outlined in the 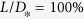 for clarity.

Figure 5a shows diametrally compressed drops of 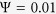 and 100 representing the incompressible and the tension-dominated regime, respectively. At small compressions, the differences between their shapes are hardly visible as seen by comparing the contours at slit width of 80% of the reference diameter D_*_. But at large compressions the almost incompressible, constant-volume 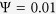 drop expands very dramatically, assuming a pronounced pancake-like shape at slit widths below about 40% of D_*_, whereas the in-plane diameter of the tension-dominated 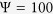 drop is only slightly larger than D_*_.

This insight is quantitatively elaborated in Fig. 5b which shows the theoretical reduced central lateral extension 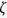 of a diametrally compressed liquid drop for 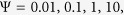 and 100. In tension-dominated drops with 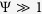, 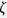 is small and barely depends on the ratio of slit width and reference drop diameter 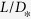 whereas in incompressible drops of 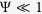, 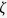 diverges at vanishing slit widths. In the incompressible limit 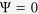, drop shape can be approximated by a jelly-doughnut shape with flat faces pressed against the walls and a rim of semi-circular cross-section, allowing 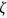 to be calculated analytically. The result (dashed line in Fig. 5b) is characterized by a 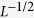 divergence in thin slits as well as a finite 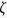 at infinitesimally small compressions, which is qualitatively consistent with the numerically obtained knee-like onset of deformation (Fig. 1b). The red shading in Fig. 5b represents the region around 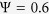 characteristic of our SPBs, showing that they are halfway between the incompressible and the tension-dominated regime.

The differential response of small- and large-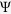 drops upon compression is even more pronounced in drops confined from all sides rather than just between two walls. This can be illustrated by a drop within a cube-shaped container (Fig. 5c). The nearly incompressible 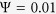 drop develops large facets pressed against container walls even at small compressions—as soon as cube edge length *L* reaches 80% of the reference diameter D_*_, facets add to 90% of the total drop area. On the other hand, in the tension-dominated 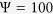 drop this happens only at 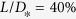, which corresponds to an eightfold larger density.

These differences are important because shape deformation similar to faceting is expected to take place in dense suspensions. Here SPBs are pressed against each other, which too can be described by the liquid-drop model (or, more generally, the combined liquid-drop/soft-ball model) much like repulsion between a star polymer and a hard wall also covers the interaction between two star polymers at small center-to-center distances^34^. Thus our coarse-grained framework may be used to interpret the structure of crystalline^35^, quasicrystalline^35^,^36^, and glassy^37^,^38^ nanocolloidal systems as well as certain aspects of their unique rheology^39^, the value of the reduced Egelstaff-Widom length 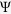 being the key material parameter.

An interesting direct application of our results addresses the morphologies of aggregated polymer-grafted nanoparticles, which include string- and sheet-like aggregates in addition to the more common spherical bulk-like clusters and dispersed solutions^20^. By using the detailed description of brush deformation in the slit we can readily analyse the lateral repulsive barrier in string-like aggregates, thereby complementing the scaling-theory^40^ and patchy-particle^41^ interpretations of their stability. Like in the Derjaguin-Landau-Verwey-Overbeek (DLVO) theory, the barrier appears because of competition of the strong but short-ranged van der Waals attraction and a repulsive interaction between them, except that the latter is due to brush deformation rather than electrostatic.

**Figure 6:**
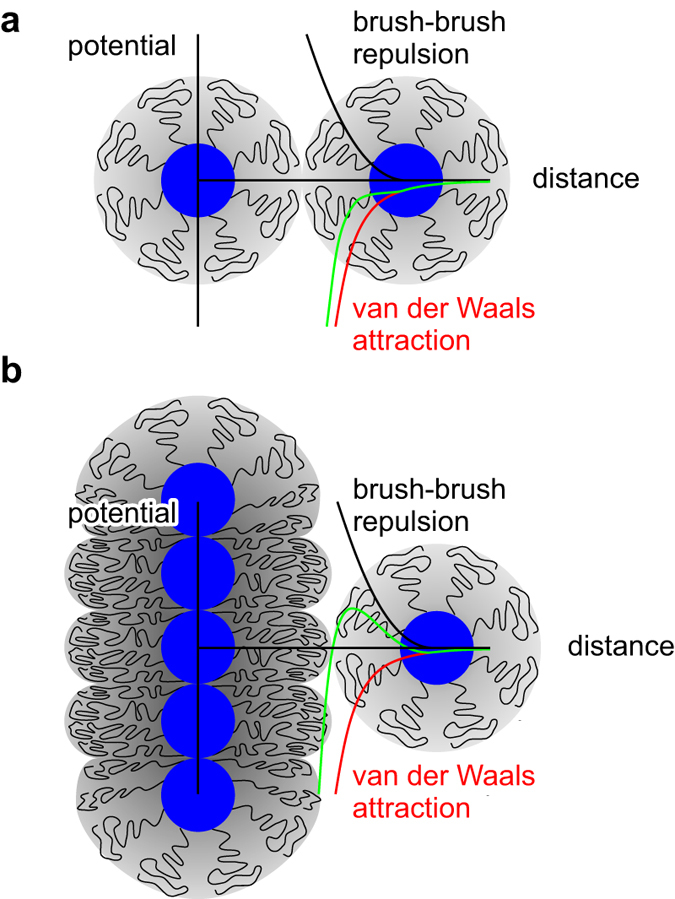
Lateral barrier in string-like aggregates. (**a**) Attraction between isolated SPBs (red line) is weakened by the elastic repulsion (black line) but in soft enough brushes the total potential (green line) is attractive. (**b**) Due to lateral extension of SPBs in a string, potential felt by an approaching SPB features a barrier and a local minimum like in the DLVO theory. The ratio of deformation energy scale *U* [equation (7)] and the Hamaker constant was tuned such that the potentials in panels a and b are monotonic and non-monotonic, respectively.

To see how this happens, consider two isolated nearby SPBs (Fig. 6a). Brought into contact by the van der Waals attraction (assumed for simplicity to be dominated by interaction between nanoparticle cores), they will interpenetrate each other as long as the effective elastic modulus of the brush is not too large. Elastic repulsion, which is approximately proportional to a power of indentation with an exponent of 2.1 (inset to Fig. 2b), will merely reduce the magnitude of attraction and the SPBs will eventually form a core-to-core packed dimer with somewhat deformed brushes. In a similar fashion, a third, fourth… SPB may approach the aggregate, and it is intuitively clear that the most favorable point of attachment is at the end of the string where the central SPB is thinnest^40^.

Let us substantiate this expectation by an argument based on the transverse extension of SPBs in the string, which are deformed as if they were confined to a slit. The extension depends on the lengthwise compression dictated by the ratio of core and brush diameters 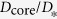. For example, if 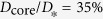 then the maximal lengthwise compression is 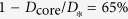, and our results suggest that the waist of a 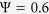 SPB is dilated by about 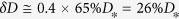 because 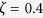 at this slit width (Fig. 1b). For another SPB approaching the string from the side, the distance upon contact is thus larger than D_*_ by 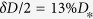 (Fig. 5b).

This may not seem much but two important factors come into play that both contribute to the stabilisation of the string. Firstly, the van der Waals interaction is a very steep function of separation and the Hamaker theory predicts that at this larger distance, the core-core attraction between an approaching SPB and a member of the string is only about 46% of the attraction between two SPBs separated by D_*_. Secondly, the confinement of all *f* chains to a pancake-shaped quasi-two-dimensional region results in an effectively larger functionality, and thus in a decrease of brush compressibility 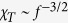. Provided that 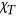 is large enough, this may lead to a total potential that is qualitatively different from that in Fig. 5a because the brush-brush repulsion produces a barrier separating the unbound state from the global minimum of core-to-core packed SPBs. If higher than the thermal energy, the barrier prevents the formation of sheet- and bulklike aggregates, thereby stabilising strings by a patently many-body mechanism. Alternatively, if the barrier is still not high enough, then aggregation of brushes will also start in a second dimension, leading to the formation of sheets and pushing the SPBs out in the direction perpendicular to the sheets. The two stabilising mechanisms mentioned above will be activated again, potentially preventing the appearance of bulk aggregates. On the other extreme, for 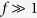 and very long chains, the chain length itself provides an effective stabilisation against coagulation, and a well-dispersed system results.

In conclusion, the elasticity of complex nanoparticles can be interpreted using coarse-grained notions from continuum mechanics, and the excellent agreement of the simulation results with the liquid-drop model provides a convincing microscopic support for a previously proposed theory of nanocolloidal crystals^42^. Our stripped-down framework can be refined in various ways, say by introducing a differential tension of the free and the contact surface of the brush or by a position-dependent Young modulus which would better correspond to the inhomogeneous monomer density, and it may be extended to non-spherical and polyelectrolyte SPBs^43^ which may behave quite differently from our self-avoiding brushes as well as to semiflexible and stiff chains^44^. In addition, the liquid-drop model could be used for the description of viscoelastic effects expected in a dynamic rather than static deformation of the SPB studied here—in this case, one would have to solve the equation of motion for the drop treated as a viscous liquid. Such a generalization may well be relevant for the rheology of suspensions of SPB-like soft colloids, e.g., star polymers^45^.

The most intriguing conceptual conclusion reached is that the deformation behaviour of our SPBs can be encoded solely by the reduced Egelstaff-Widom length 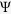, a dimensionless quantity based on the product of surface tension and compressibility which is known to have a very similar value in many simple liquids^26^. Moreover, the value of 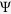 is universal, i.e., independent of the number of chains in the brush and their degree of polymerisation. These findings call for further verification. In turn, it would be interesting to see whether our model also applies to other polymeric nanocolloidal particles such as dendrimer and diblock copolymer micelles and if so, how does the reduced Egelstaff-Widom length depend on the macromolecular architecture.

## Methods

### Molecular dynamics simulations

Our spherical brush consists of bead-and-spring chains terminally grafted onto a small colloidal particle such that the anchor points are fixed and distributed uniformly across the particle. The main parameters of the SPB are the number of chains *f* and the number of monomers per chain *N*_*c*_; colloid diameter is 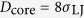 where 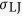 is the monomer diameter. Like in a related study^46^, steric monomer-monomer and monomer-colloid interaction is described by the Weeks-Chandler-Andersen (WCA) repulsion^47^ and the bonds are modelled by the finite extensible nonlinear elastic (FENE) potential tuned such that the bonds do not cross. Our implicit-solvent scheme and this particular choice of interactions are both consistent with the good solvent conditions studied here. The parameters of the FENE potential used were 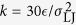 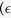 being the strength of the WCA repulsion) and 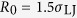 as suggested in ref. 48. For numerical convenience, the walls are represented by an external Yukawa potential^49^.

Two-step velocity-Verlet molecular dynamics (MD) simulations at a constant temperature 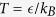 were used to find the equilibrium SPB configurations in several independent simulation runs [seven for all SPBs except for three of the *N*_*c*_ = 50 SPBs: *f* = 40 (four) and 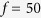 and 60 (six)]. Each run involved a gradual decrease of slit width from a large value where the walls did not affect the SPB down to 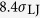. An isolated SPB was initially equilibrated for 5.5 × 10^7^ time steps (equal to 
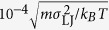
 where *m* is the monomer mass) and then at any given slit width the SPB was equilibrated for 2 × 10^6^ steps followed by 8 × 10^6^ measurement steps.

### Self-consistent field theory

The self-consistent field (SCF) theory was implemented on a cubic lattice using the Scheutjens-Fleer scheme^46^,^50^. We chose to represent the walls by a hard rather than Yukawa potential like in MD simulation. This allowed us to analyse the effect of confinement and the SPB geometry as transparently as possible and facilitated comparison with the liquid-drop and soft-ball models.

### Continuum mechanics models

In both models, walls were represented by hard constraints so as to study the model in as simple a geometry as possible; also neglected is the SPB core. The equilibrium shape of the SPB represented by the liquid drop was obtained using the Surface Evolver package^27^ where the drop is represented by a triangulated surface. The shape was found by relaxation mimicking overdamped motion of the vertices to minimize the combined bulk and surface energy [equation (3)]; the typical number of nodes on the surface was about 3000.

The soft-ball model was solved by minimizing equation (8) using the finite-element method implemented within the FreeFEM++ package^30^. The displacement field was computed on a 3D mesh of about 40000 nodes using a multifrontal LU factorization solver UMFPACK. At any slit width, equilibrium was achieved using Newton-Raphson iteration scheme. To enforce the hard-wall constraints, the magnitude of the parabolic wall potential was gradually increased until the corresponding energy penalty was negligible.

## Additional Information

**How to cite this article**: Riest, J. *et al.* Elasticity of polymeric nanocolloidal particles. *Sci. Rep.* **5**, 15854; doi: 10.1038/srep15854 (2015).
